# Evolution, Expression Profile, Regulatory Mechanism, and Functional Verification of EBP-Like Gene in Cholesterol Biosynthetic Process in Chickens (Gallus Gallus)

**DOI:** 10.3389/fgene.2020.587546

**Published:** 2021-01-14

**Authors:** Keren Jiang, Zheng Ma, Zhang Wang, Hong Li, Yanbin Wang, Yadong Tian, Donghua Li, Xiaojun Liu

**Affiliations:** ^1^College of Animal Science, Henan Agricultural University, Zhengzhou, China; ^2^School of Life Sciences and Engineering, Foshan University, Foshan, China; ^3^Henan Innovative Engineering Research Center of Poultry Germplasm Resource, Zhengzhou, China; ^4^International Joint Research Laboratory for Poultry Breeding of Henan, Zhengzhou, China

**Keywords:** EBP-like, liver, cholesterol synthesis, chicken, estrogen

## Abstract

The emopamil binding protein (EBP) is an important enzyme participating in the final steps of cholesterol biosynthesis in mammals. A predictive gene *EBP-like*, which encodes the protein with a high identity to human EBP, was found in chicken genome. No regulatory mechanisms and biological functions of *EBP-like* have been characterized in chickens. In the present study, the coding sequence of *EBP-like* was cloned, the phylogenetic trees of EBP/EBP-like were constructed and the genomic synteny of *EBP-like* was analyzed. The regulatory mechanism of *EBP-like* were explored with *in vivo* and *in vitro* experiments. The biological functions of *EBP-like* in liver cholesterol biosynthetic were examined by using gain- or loss-of-function strategies. The results showed that chicken *EBP-like* gene was originated from a common ancestral with Japanese quail *EBP* gene, and was relatively conservative with *EBP* gene among different species. The *EBP-like* gene was highly expressed in liver, its expression level was significantly increased in peak-laying stage, and was upregulated by estrogen. Inhibition of the *EBP-like* mRNA expression could restrain the expressions of *EBP-like* downstream genes (*SC5D*, *DHCR24*, and *DHCR7*) in the cholesterol synthetic pathway, therefore downregulate the liver intracellular T-CHO level. In conclusion, as substitute of *EBP* gene in chickens, *EBP-like* plays a vital role in the process of chicken liver cholesterol synthesis. This research provides a basis for revealing the molecular regulatory mechanism of cholesterol synthesis in birds, contributes insights into the improvement of the growth and development, laying performance and egg quality in poultry.

## Introduction

As an important lipid molecule, cholesterol plays essential roles in growth and differentiation of eukaryotic cells, as well as in regulating the properties of plasma membranes in cells by affecting membrane fluidity, phase behavior, thickness, and permeability (Crockett, 1998; Simons and Ikonen, 2000). Cholesterol is also a vital precursor for hormones, bile acids, and lipoproteins production.

The process of cholesterol uptake can be divided into dietary pathway and endogenous pathway. In mammal, the cellular cholesterol requirements can be met by either internalization of low density lipoproteins (LDL) or *de novo* synthesis in the endoplasmic reticulum (ER) (Colbeau et al., 1971; Brown et al., 1981; Liscum and Underwood, 1995). In humans, the liver and ileum are the primary sites of cholesterol biosynthesis (Dietschy and Wilson, 1970). Cholesterol synthesis in hepatocytes can be divided into three main stages catalyzed by functional specific enzymes (Sakakura et al., 2001; Lorbek et al., 2013). At the first stage, Acetyl coenzyme A was catalyzed to produce HMG-CoA by sulfur lyase and other enzymes, then catalyzed by HMGCR (3-Hydroxy-3-methylglutaryl-CoA reductase) to form mevalonate (MVA), which will be converted into isoprene pyrophosphate (IPP) during enzymatic reactions such as phosphorylation and decarboxylation. At the second stage, IPP was converted into squalene by phosphorylation, decarboxylation, dehydroxylation, and multi-step condensations. At the final stage, the product of the previous step was catalyzed by cyclase and oxygenase into lanosterol, which was then reacted in multiple steps to produce cholesterol (Ačimovič and Rozman, 2013). The emopamil binding protein (EBP) plays a key role in the final stage by acting as a D8-D7 sterol isomerase which converts Cholest8(9)-en-3beta-ol (8,9 choletenol) into cholest-7-en-3beta-ol (lathosterol).

While much is known about cholesterol biosynthesis and its regulatory process in mammals (Goldstein and Brown, 1990; Horton et al., 2002a), little is known about it in chickens. Differing from mammals, chickens absorbing dietary cholesterol by hydrolyzing it into free cholesterol, which can be converted into cholesterol ester in small intestine, then transported to circulation through lymphatic system. Because the lymphatic system in birds is not as well developed as that in mammals, the majority of the cholesterol needed by birds is self-synthesized in liver (Bensadoun and Rothfeld, 1972). There is about 300 mg cholesterol needed every day for a laying hen in order to produce the eggs, about 200 mg cholesterol will be deposited in egg yolk and the rest of the cholesterol will be discharged into intestine and will be converted into steroids and vitamin D to support the nutrition requirements (Naber, 1983). Therefore, cholesterol biosynthesis is an important process in avian, especially for laying hens. Up to now, studies on the regulations of cholesterol synthesis in chickens mainly focus on the exogenous factors. For instance, previous studies found that increasing dietary protein level can promote the liver cholesterol synthesis, while inhibiting the total plasma cholesterol level (Yeh and Leveille, 1972); the low-energy diet affects genes involved in the cholesterol synthesis (Jehl et al., 2019); high cholesterol diet, chitosan supplementation, green tea powder supplementation and application of probiotic can inhibit the total blood cholesterol level; while stilbestrol feeding and estrogen treatment can significantly increase plasma cholesterol concentration as well as liver cholesterol level (Sakakida et al., 1963; Klimis-Tavantzis et al., 1983; Adriani et al., 2018; Chen et al., 2020; Kumalasari et al., 2020). However, few studies have focused on the endogenous cholesterol biosynthetic process in chickens.

To gain insights into the synthesis and regulatory mechanism of cholesterol in chickens, amino acid sequences of the enzymes involved in human cholesterol biosynthesis were used as queries to search for their analogs against chicken protein database (Gallus_gallus-6.0, ensemble release 97). It was noticed that, among all enzymes that involved in the human cholesterol biosynthesis pathway, only EBP is missing in chicken genome. However, a predictive gene (LOC100857622) in chickens, named as 3-beta-hydroxysteroid-Delta(8), Delta(7)-isomerase-like (*EBP-like*), showed a high positive sequence identity with the human *EBP*. To the best of our knowledge, the biological function and regulatory mechanism of *EBP-like* gene in chickens have not been characterized. Therefore, the objectives of this work are to (1) analysis the evolutionary history of *EBP-like*, (2) investigate its expression pattern and reveal its regulatory mechanism in chicken liver, and (3) explore its biological functions in chicken cholesterol biosynthesis.

## Materials and Methods

### Ethics Approval

Animal experiments were approved by the Institutional Animal Care and Use Committee (IACUC) of Henan Agricultural University Zhengzhou, P.R. China and performed following the guidelines of National Institutes of Health Guide for the Care and Use of Laboratory Animals (NIH Publications No. 8023, revised 1978).

### Animal and Sampling

Lushi blue-shelled chicken, one of the Chinese native local breeds, was used in this study. All chickens were raised in cages in the same environmental conditions with *ad libitum* access to food and water. Twelve hens were randomly selected from each of the different developmental stages at 5, 15, 20, 30, and 35 weeks old. The birds were sacrificed and tissues including lung, liver, spleen, heart, kidney, glandular stomach, pectoral muscle, pancreas, duodenum, leg muscle, abdominal fat, and ovary were harvested, immediately snap-frozen in liquid nitrogen and stored at −80°C until use.

To study the regulatory mechanism of *EBP-like* gene, 40 hens at the age of 10 weeks old were randomly divided into four groups with 10 birds in each group. The birds in the first three groups were intramuscularly injected in the pectoral muscles with 0.5, 1.0, and 2.0 mg/kg of 17β-estradiol (Sigma, St. Louis, MO, United States) dissolved in olive oil, respectively (Rusinol and Bloj, 1989; Zhao et al., 2017). The birds in the control group were intramuscularly injected with simply the same amount of olive oil. All birds from the four groups were sacrificed at 12 h post-injection and the liver tissues were collected and stored as mentioned above.

### Cloning and Sequence Bioinformatics Analysis of Chicken EBP-Like Gene

The human EBP amino acid sequence was used as a query to search in chicken protein database (Gallus_gallus-6.0, ensemble release 97) using program BLASTn and BLASTx on NCBI^1^. According to the predicted chicken *EBP-like* gene sequence (LOC100857622) on GenBank, PCR primers were designed using NCBI Primer-BLAST (Table 1). The PCR products were sequenced by Beijing Genomics Institute (BGI). Amino acid sequences of EBP-like in chickens and EBP-like/EBP in other species (Supplementary Table 1) were retrieved from the NCBI protein database.

**TABLE 1:** Primers for EBP-like cloning.

**Gene name**	**Sequence (5′–3′)**	**TM (°C)**	**Product (bp)**
EBP-like	F: GAGCGACCAGGAGACTCGC	56	765
	R: AGGTCCGCACGTAGAGGG		

*F, forward primer; R, reverse primer.*

To reveal the evolutionary relationship of chicken *EBP-like* with *EBP/EBP-like* in other species, the rooted phylogenetic time-tree was constructed by using the maximum likelihood method on Molecular Evolutionary Genetics Analysis version 7.0 (MEGA 7.0) with default parameters (Kumar et al., 2016). Zebrafish EBP (accession number NC_007134.7) was used as an outgroup. The evolutionary time-tree was generated based on the coding sequences of *EBP/EBP-like* by using the RelTime method (Tamura et al., 2012). Divergence times for branching points in the topology were calculated based on the JTT matrix-based model (Jones et al., 1992). In order to classify EBP/EBP-like in different species, the unrooted phylogenetic tree was constructed using the maximum likelihood method on MEGA 7.0 based on amino acid sequence alignments generated from Clustal W (Strimmer and Von Haeseler, 1996). The reliability of the tree was assessed using 1,000 bootstrap replicates. The numbers at each clade represent bootstrap support values were given as percentages (Tian et al., 2018; Yang et al., 2019). Percentage amino acid sequence identity and sequence similarity were determined using the BLOSUM62 matrix algorithm (Styczynski et al., 2008). The Genomicus v99.01 online program^2^ was employed for consensus conserved genomic synteny analysis. The functional domain of chicken EBP-like protein was analyzed on SMART website^3^.

### RNA Extraction, cDNA Synthesis, and Quantitative RT-PCR

Total RNA was extracted from the tissues and cultured cells using TRIzol^®^ reagents following the manufacturer’s manual (Invitrogen, Carlsbad, CA). The RNA concentration and purity were assessed using NanoDrop2000 spectrophotometer (Thermo Fisher Scientific, Wilmington, DE, United States) and RNA integrity was analyzed by denatured agarose gel electrophoresis. The RNA samples with OD260/280 ratios above 1.8 and the 28S and 18S bands with brightness in denatured agarose gel was selected for further use. The PrimeScript^TM^ RT reagent Kit with gDNA Eraser was used to reverse-transcribe RNA samples into cDNA (TaKaRa, China), the cDNA was stored at −20°C until use. The relative expression levels of mRNAs were detected by Real-time PCR (RT-PCR) and β-actin were used as internal control. The RT-PCR was performed with Power SYBR Green PCR Master Mix (TaKaRa, China) using the LightCycler 96 Real-Time PCR System (Roche, Switzerland). The RT-PCR amplification procedure was as follows: 95°C for 3 min; 35 cycles of 95°C for 30 s, 60°C for 30 s, 72°C for 20 s, and an extension for 10 min at 72°C. Relative expression levels of mRNAs were determined using the 2^–ΔΔ*Ct*^ method as provided by the manufacturer (Livak and Schmittgen, 2001). The sequences of RT-PCR primers used in this study were shown in Table 2.

**TABLE 2:** RT-PCR primers.

**Gene name**	**Transcript ID**	**Sequence (5′–3′)**	**Product (bp)**
EBP-like	XM_015274577.2	F: GTCTCCCTCGGACAGCTCTA	111
		R: CCCCACGAAGTACACCCAAA	
HMGCR	NM_204485.2	F: ATGTCAGGAGTGCGACAACT	140
		R: CGTCCTTCACGACTCTCTCG	
SC5D	XM_015298176.1	F: CTTAGAGAACCAGGTGCAACG	114
		R: TACAGTTTGCTGTAGCCCCG	
DHCR7	NM_001199490.1	F: AGGACTGATAGCCGGGCA	140
		R: ACCAGTCTACCTCCCATGCT	
DHCR24	NM_001031288.1	F: TCTTCGACGTGTACTACCAGC	268
		R: TCCACGCGAACAACCTGTCT	
Apo-VLDLII	NM_205483.2	F: CAATGAAACGGCTAGACTCA	108
		R: AACACCGACTTTTCTTCCAA	
β-actin	NM_205518.1	F: GAGAGAAGATGACACAGATC	116
		R: GTCCATCACAATACCAGTGG	

*F, forward primer; R, reverse primer.*

### Vector Construction and siRNA Oligonucleotide Synthesis

In order to generate *EBP-like* overexpression vectors, the coding sequences of *EBP-like* with *Hin*dIII restriction site (AAGCTT) and the Kozark sequence (GCCACC) on the 5′ end, and *Bam*HI restriction site (GGATCC) on the 3′ end were synthesized by Shanghai GenePharma (Shanghai, China), and cloned into the pcDNA3.1-EGFP vector (Invitrogen^TM^, United States) using restriction endonuclease *Hin*dIII and *Bam*HI. The products were sequenced by Beijing Genomics Institute (Beijing, China). In order to obtain the *EBP-like* specific RNA interference fragment, *EBP-like* siRNA fragments (si-*EBP-like*) with FAM fluorescent labels were designed and synthesized by Shanghai GenePharma (China). The detailed information of the siRNA sequence is shown in Table 3.

**TABLE 3:** Si-RNA sequences.

**Si-RNA name**	**Sense sequence (5′–3′)**	**Antisense sequence**
Si-NC	UUCUCCGAACGUGUCACGUTT	ACGUGACACGUUCGGAGAATT
Si-EBP-like	CUGGAAGGAAUACGCCAAATT	UUUGGCGUAUUCCUUCCAGTT

### Cell Culture, Treatment, and Transfection

Chicken hepatocellular carcinoma cell line (LMH) was obtained from Prof. Zhang Li (Guangdong Ocean University, China) and was grown in Dulbecco’s modified Eagle’s medium (DMEM)(GIBCO, United States) supplemented with 10% FBS and penicillin (100 U/mL)/streptomycin (100 mg/mL). Before estrogen treatment, LMH were seeded in 12-well plate at a density of 3.0 × 10^5^ cells/well, and cultured in a humidified incubator at 37°C with 5% CO_2_. When the cells reached 80–90% confluence, they were serum-free starved for 6 h and randomly divided into four groups with six replicates in each group. Cells in the first 3 groups were treated with 25, 50, and 100 nM 17β-estradiol dissolved in ethyl alcohol, respectively. Cells in control group were treated with the same amount of solvent only. After incubation for 12 h, the cells were washed with PBS for 3 times, collected with TRIzol^®^ reagents (Takara, Kyoto, Japan). For the transfection assay, the cells were serum-starved for 6 h, after reached 80% confluence, the cells were transfected with pcDNA3.1-*EBP-like* (600 ng) or si-*EBP-like* (6 μl) in serum-free DMEM medium using TurboFect transfection reagent (Invitrogen, Thermo Fisher Scientific, United States). The medium was changed after 6 h of transfection. The transfection experiment was performed in triplicate and repeated at least three times. At 48 h post-transfection, cells were harvested and lysed in passive lysis buffer (Promega, United States) for further use.

### Test of Intracellular Total Cholesterol Level

The cell culture medium was removed, and the chicken hepatocellular carcinoma cells were washed with PBS for 3 times before incubated with 0.25% (w/v) Trypsin, 0.53 mM EDTA solution at 37°C for 2–5 min. After centrifuging cells at 1,100 rpm, the supernatant was discarded and 500 μl PBS was added. Cell breakage was accomplished and the intracellular total cholesterol (T-CHO) was determined using cholesterol detection kit (Nanjing Jiancheng Bioengineering Institute, China) according to the manufacturer’s instruction. An appropriate amount of fresh cellular compounds was used to measure total protein concentration to normalize T-CHO level using BCA Protein Quantification Kit (Applygen, Beijing, China). All experiments were performed in triplicate, and independently repeated three times. The standard curves for intracellular T-CHO and total protein measurement were calculated using the internal standards following the manufacturer’s instructions.

### Statistical Analysis

Statistical analysis was performed by one-way ANOVA followed by Dunnett’s test using SPSS version 20.0 (IBM, Chicago, IL, United States). Graphics were drawn using GraphPad Prism 6 (Graphpad Software, San Diego, CA, United States) and the results are presented as the mean ± SD. *P* < 0.05 was considered statistically significant and *P* < 0.01 was considered highly statistically significant.

## Results

### Sequence Analysis and Cloning of Chicken EBP-Like

The human EBP amino acid sequence (NP_006570.1) was used as a query to search in chicken protein database, the protein XP_015130063.1 showed up with a positive identity of 65% to the amino acids of human EBP protein (Supplementary Figures 1A,B). The corresponding mRNA of the protein XP_015130063.1 is a predictive gene, named as LOC100857622 3-beta-hydroxysteroid-Delta(8), Delta(7)-isomerase-like (*EBP-like*) on NCBI. According to the coding sequence of chicken *EBP-like*, a protein consisted of 218 amino acid residues was deduced (Supplementary Figure 2B). A conserved EBP fingerprint domain was identified in chicken EBP-like protein sequence starts from 28aa and ends at 206aa (Supplementary Figure 2C). A total of 765 bp sequence including full-length of coding sequence region and the approximate 50 bp on each end of *EBP-like* was obtained by PCR using chicken liver cDNA as template. Sequence alignment showed that the *EBP-like* coding sequence were successfully cloned with 100% identity to the *EBP-like* coding sequence predicted on NCBI GenBank (Supplementary Figure 2A).

### Evolutionary Relationship and Phylogenetic Analysis of Chicken EBP-Like

**FIGURE 1:**
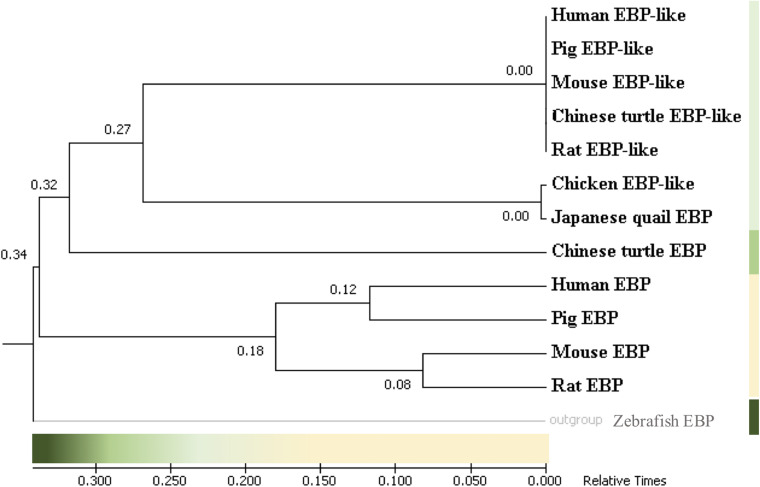
Phylogenetic relationship analysis of *EBP*/*EBP-like*. Time-scale tree for the evolution of *EBP*/*EBP-like* gene. The tree was rooted by using zebrafish EBP as an outgroup. The relative gene duplication times are shown in × 1,000 millions years ago.

In order to study the origin and the evolutionary relationship of chicken EBP-like and EBP/EBP-like in other species, the protein sequences of EBP/EBP-like among eight representative species, including mammals (human, mouse, rat, pig), avian (chicken, Japanese quail), reptile (Chinese turtle), and fish (zebrafish), were downloaded from NCBI database. A rooted evolutionary tree was constructed. Zebrafish EBP sequence was used as an outgroup to locate the deepest branch (i.e., duplication of the primordial *EBP*/*EBP-like*), the relative divergence times between clusters were estimated (Figure 1). The duplication of Chinese turtle *EBP* gene occurred approximately 20 million years after the divergence of zebrafish *EBP*. Around 50 million years after the duplication of Chinese turtle *EBP* gene, two sub-clusters of *EBP-like* genes were estimated diverged. The first sub-cluster includes chicken *EBP-like* and Japanese quail *EBP* gene, which were duplicated slightly earlier than the second sub-cluster. The second sub-cluster includes *EBP-like* gene in human, pig, mouse, rat, and Chinese turtle. The duplication of *EBP* genes in human, pig, mouse, and rat is relatively late, occurred about 90 million years after the duplication of *EBP-like* genes (Figure 1). The results indicate that chicken *EBP-like* gene emerged at a relative early time along with other *EBP-like* genes in reptile and mammals. Chicken *EBP-like* gene were grouped into a same cluster with Japanese quail EBP gene, suggesting that they are originated from one common ancestral gene.

**FIGURE 2:**
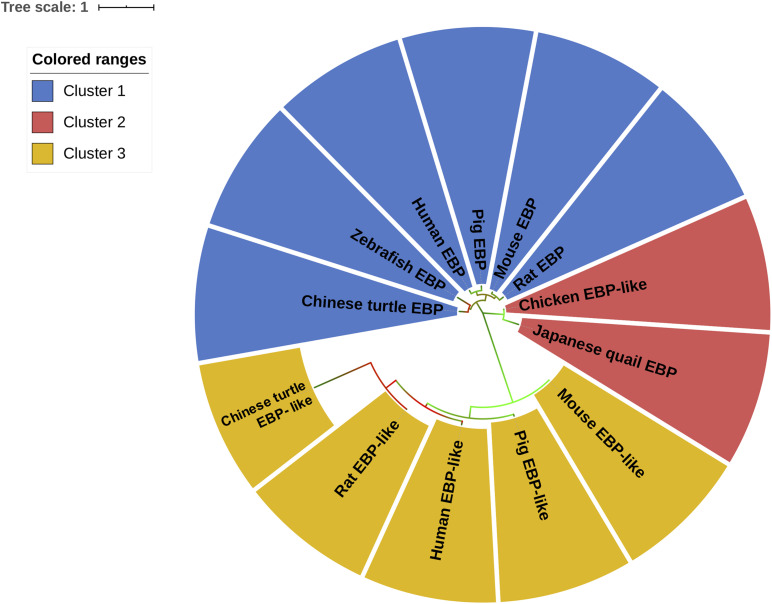
Molecular phylogenetic analysis of *EBP*/*EBP-like*. The unrooted tree is drawn to scale, with branch lengths measured in the number of substitutions per site. Chicken *EBP-like* gene and *EBP*/*EBP-like* genes from other species were divided into three clusters according their protein sequences similarities. Different clusters are distinguished by colors.

In order to cluster EBP/EBP-like in different species, an unrooted evolutionary tree was constructed to classify all of the sequences based on sequence similarity. The results suggest that EBP/EBP-like can be grouped into three main clusters (Figure 2). The EBPs in Chinese turtle, zebrafish, human, pig, mouse, and rat were grouped into the first cluster (blue). The two internal sub-branches in cluster 1 separated fish/reptile (zebrafish, Chinese turtle) EBP and mammalian (human, mouse, rat, pig) EBP. The EBP-likes in Chinese turtle, human, pig, mouse, and rat were clustered into the third branch (yellow). The phylogenetic tree separated EBP-like from EBP, with chicken EBP-like as an exception, which has been clustered into the second group (red) with Japanese quail EBP (Figure 2). Although chicken EBP-like and Japanese quail EBP were clustered into a distinct group, they shared a certain level of sequence similarities with the EBPs from other species.

### Conserved Synteny for the Genomic Region of EBP/EBP-Like Neighboring Genes

**FIGURE 3:**
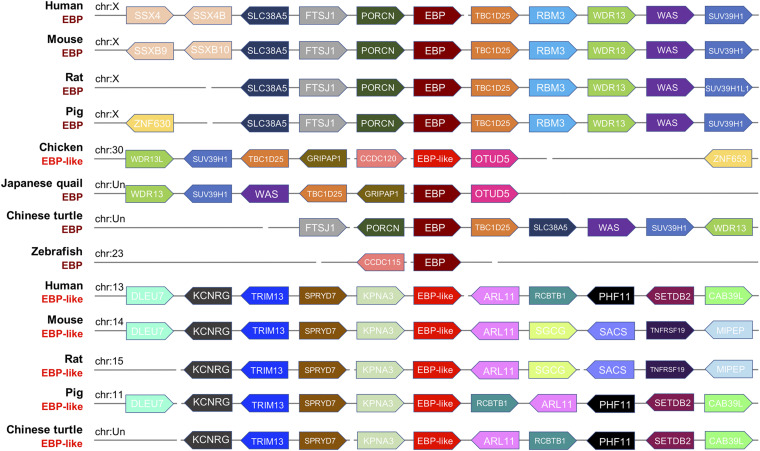
Syntenic relationship of gene loci mapped around the *EBP*/*EBP-like* gene in selected species. The gene name, species and chromosome distribution are listed on the left. Every pentagon refers to a gene, and the same color in columns means the same gene existing in the corresponding species. The direction represents DNA strand for gene transcription, right refers to sense strand, left refers to antisense strand.

To further investigate whether the *EBP-like* in chickens is the orthologous of *EBP* gene in other species, a syntenic analysis of the *EBP*/*EBP-like* neighboring genes was performed in eight representative genomes including mammals (human, mouse, rat, pig), avian (chicken, Japanese quail), reptile (Chinese turtle), and fish (zebrafish). The results indicated a high degree of conservation of synteny among *EBP*s in mammals, which are all linked on chromosome X. The *EBP* neighboring genes in Chinese turtle and zebrafish have a relative poor conservative synteny compare with both mammalian and avian *EBPs* (Figure 3). *EBP-likes* in human, mouse, rat, pig, and Chinese turtle locate at distinct chromosomes, but are with highly conserved neighboring genes. The neighboring genes of chicken *EBP-like* and the neighboring genes of Japanese quail *EBP* were distributed in a highly conservative manner, meanwhile, chicken *EBP-like* and Japanese quail *EBP* share a relative low conservation of synteny with mammal *EBP*s. However, chicken *EBP-like* showed no conservative synteny comparing with *EBP-like* in other species (Figure 3).

### Spatiotemporal Expression Profiles of EBP-Like Gene

**FIGURE 4:**
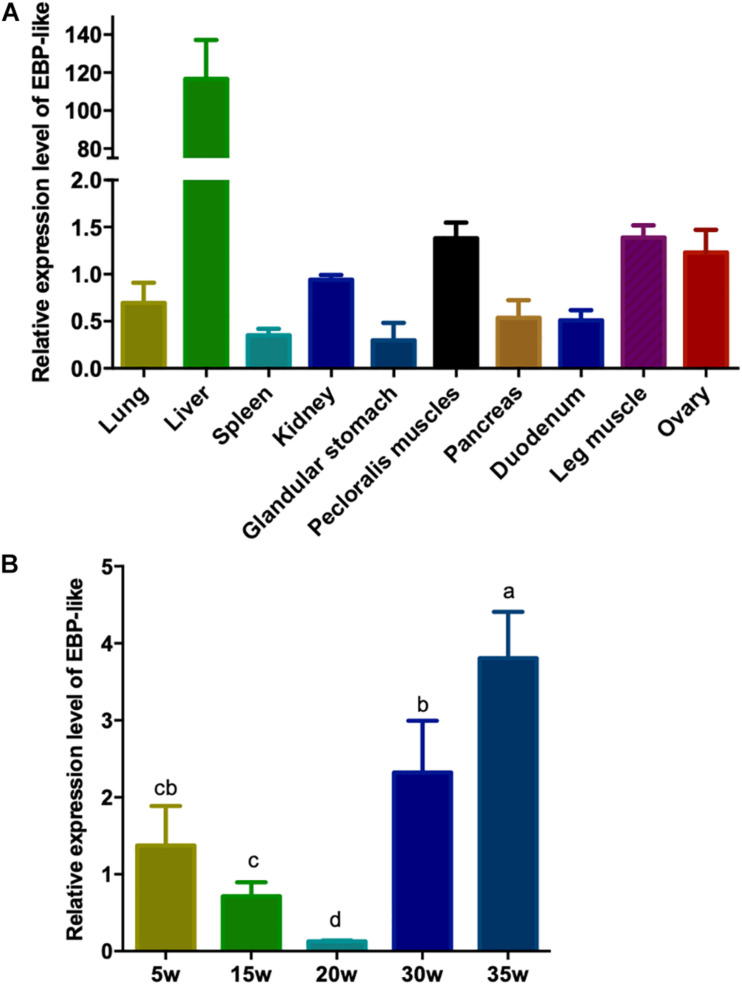
*EBP-like* spatiotemporal expression profiles. **(A)** The relative expression level of *EBP-like* in different tissue of laying hens (*n* = 6). **(B)** The relative expression level of *EBP-like* in liver tissue of chicken at different developmental stages (*n* = 6). Note: all data are presented as mean ± SD (*n* = 4–6), groups with different letters are significantly different (*P* < 0.05), groups with same letters have no significant difference (*P* > 0.05).

In order to study the expression profile of *EBP-like* gene in different tissues of chicken, the relative expression levels of *EBP-like* gene in lung, liver, spleen, kidney, glandular stomach, pectoral muscle, pancreas, duodenum, leg muscle, and ovary of peak-laying chicken (30-weeks-old) were detected. The results showed that *EBP-like*, with a very low expression level in other tissues, was significantly highly expressed in liver tissue (*P* < 0.01) (Figure 4A). Furthermore, the expression levels of *EBP-like* in chicken liver at different developmental stages were studied. The results showed that, the expression level of *EBP-like* decreased from 5 to 20 weeks, followed by a significantly increase from 20 to 35 weeks (*P* < 0.05) (Figure 4B). It indicated that the expression of *EBP-like* increased with the sexual maturity of hens.

### Regulation of Estrogen on the Expression of Chicken EBP-Like Gene *in vitro* and *in vivo*

**FIGURE 5:**
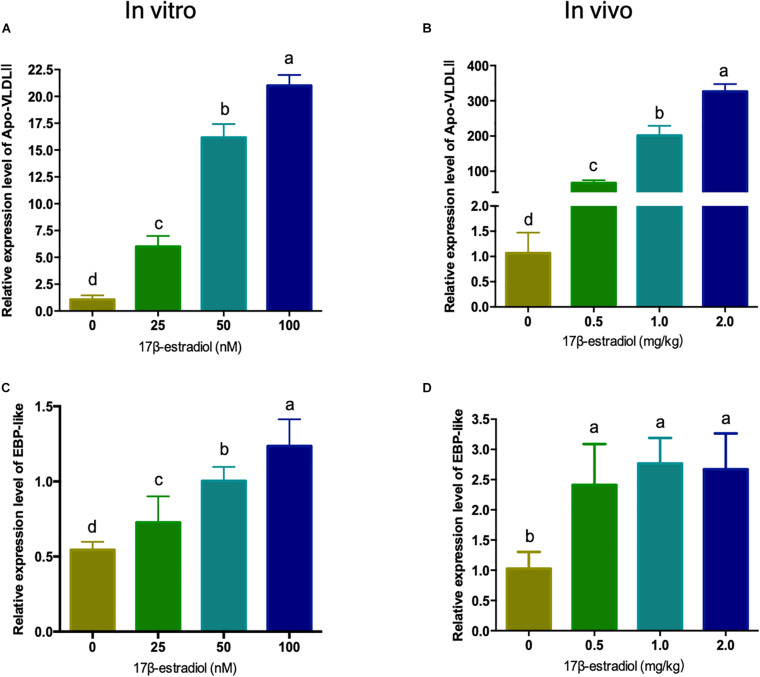
Effects of estrogen on the expression of chicken *EBP-like* gene *in vitro* and *in vivo*. **(A)** Effects of different concentrations of estrogen on the expression of *Apo-VLDLII* gene in chicken hepatocellular carcinoma cells. **(B)** Effects of different concentrations of estrogen on the expression of *Apo-VLDLII* gene in chicken liver tissues (*n* = 6). **(C)** Effects of different concentrations of estrogen on the expression of *EBP-like* gene in chicken hepatocellular carcinoma cells. **(D)** Effects of different concentrations of estrogen on the expression of *EBP-like* gene in chicken liver tissues (*n* = 6). Note: all data are presented as mean ± SD (*n* = 4–6), groups with different letters are significantly different (*P* < 0.05), groups with same letters have no significant difference (*P* > 0.05).

Estrogen is generally believed to be a major factor regulating the expression level of lipid metabolism related genes in the liver of hens. In order to explore the regulating mechanism of *EBP-like*, the mRNA expression levels of chicken *EBP-like* were evaluated both *in vitro* and *in vivo* with different doses of 17β-estradiol treatment. As a marker gene of estrogen treatment, *Apo-VLDLII* (Apovitellenin 1) expression level was detected. The expression level of *Apo-VLDLII* showed a significant dose-dependent increased in both chicken liver and LMH cells (*P* < 0.05), which indicates that the estrogen treatment model was successfully constructed with effective 17β-estradiol treatment both *in vitro* and *in vivo* (Figures 5A,B). The *EBP-like* expression level showed a significant dose-dependent increased response to the estrogen *in vitro* (*P* < 0.05) (Figure 5C). Meanwhile, the expression levels of *EBP-like* were significantly increased with the treatment of 17β-estradiol *in vivo* (*P* < 0.05), however, the effects showed no significant differences among different doses of estrogen treatment groups (Figure 5D).

### Effects of EBP-Like on the Cholesterol Synthesis

**FIGURE 6:**
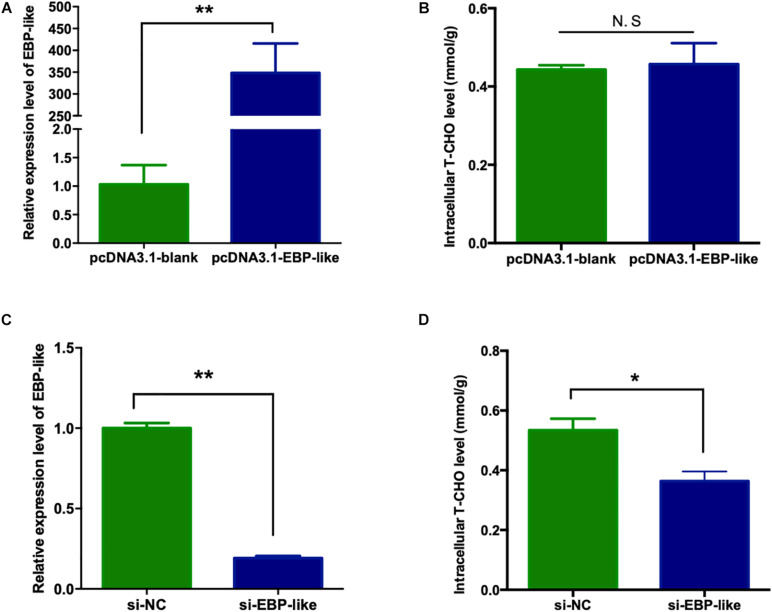
Effect of *EBP-like* gene on intracellular cholesterol level. **(A)** Expression level of *EBP-like* gene after transfection with pcDNA3.1-EBP-like vector. **(B)** Effect of *EBP-like* overexpression on liver intracellular cholesterol level. **(C)** Expression level of *EBP-like* gene after transfection with si-EBP-like vector. **(D)** Effect of *EBP-like* interference on liver intracellular cholesterol level. All data are presented as mean ± SD (*n* = 4–6). *P < 0.05, **P < 0.01. N.S represents for no significant difference (*P* > 0.05).

In order to explore the effect of *EBP-like* gene on cholesterol synthesis in liver of chicken, the overexpression vector of chicken EBP-like, pcDNA3.1-EGFP-EBP-like, was successfully constructed (Supplementary Figure 2D), and was further verified by sequencing. LMH cells were cultured and transfected with pcDNA3.1-EGFP-blank and pcDNA3.1-EGFP-EBP-like vector, respectively. At 24 h post-transfection, green fluorescence were observed in the cells, inferring that the vectors were successfully transfected into LMH cells. Comparing with the control group (transfected with pcDNA3.1-blank), the relative expression level of EBP-*like* increased over 300 times after cells transfected with pcDNA3.1-*EBP-like* vector (*P* < 0.01) (Figure 6A), which indicates that *EBP-like* was remarkably overexpressed in the transfected cells. Meanwhile, the relative expression levels of *EBP-like* significantly decreased after 24 h transfection with si-*EBP-like* in LMH cells, comparing with the si-NC (negative control) group (*P* < 0.01) (Figure 6C), indicating that the *EBP-like* interference is effective.

The intracellular T-CHO levels were measured in the above cells. No significant difference was found on the intracellular T-CHO levels when *EBP-like* gene was overexpressed in comparison with the control group (*P* > 0.05) (Figure 6B). However, intracellular T-CHO level was significantly decreased in comparison with the control group when *EBP-like* gene was knocked down by RNA interference (*P* < 0.05) (Figure 6D).

### Effects of EBP-Like on the Expression Level of Genes Related to Cholesterol Synthesis

**FIGURE 7:**
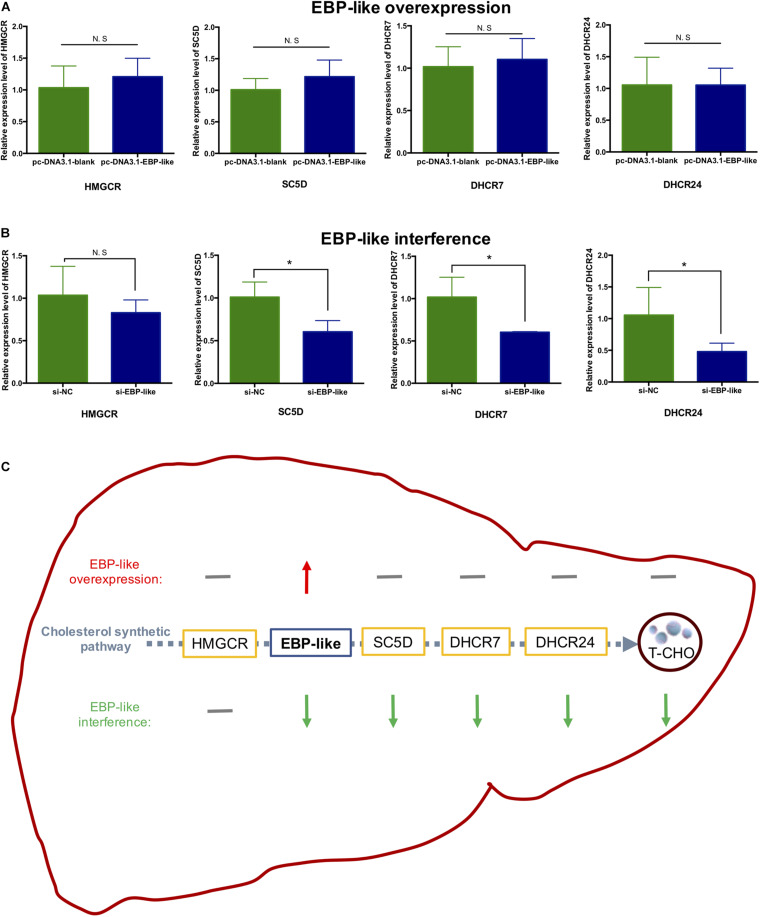
Effect of *EBP-like* on the expression level of genes related to cholesterol synthesis. **(A)** Effect of *EBP-like* overexpression on the expressions of *HMGCR*, *SC5D*, *DHCR7*, and *DHCR24*. **(B)** Effect of *EBP-like* interference on the expressions of *HMGCR*, *SC5D*, *DHCR7*, and *DHCR24*. **(C)** Effects of *EBP-like* overexpression and interference on the expressions of cholesterol synthesis pathway genes and the liver intracellular cholesterol (T-CHO) level. Note: all data are presented as mean ± SD (*n* = 4–6). *P < 0.05. Red upward arrow represents for upregulated; green downward arrow represents for downregulated; gray horizontal line represents for no significant difference.

The expression levels of upstream and downstream genes of *EBP-like* in the cholesterol synthetic pathway were detected, and the results showed that overexpression of *EBP-like* had no effects on the expression levels of *HMGCR*, *SC5D* (sterol-C5-desaturase), *DHCR7* (7-dehydrocholesterol reductase) and *DHCR24* (24-dehydrocholesterol reductase) (*P* > 0.05) (Figure 7A). However, interference of the *EBP-like* expression could significantly decrease the expression levels of *EBP-like* downstream genes *SC5D*, *DHCR7*, and *DHCR24* (P < 0.05), but had no significant effect on the expression of *HMGCR*, an upstream gene of *EBP-like* in the cholesterol synthetic pathway (*P* > 0.05) (Figure 7B). The overall effects of EBP-like on the expressions of cholesterol synthesis genes and the liver intracellular T-CHO level was shown in Figure 7C.

## Discussion

Emopamil binding protein (EBP), also known as 3-beta-hydroxysteroid-Delta(8), Delta(7)-isomerase, or cholestenol delta-isomerase, is a cholesterol biosynthesis enzyme localized in the ER and the nuclear membrane (Dussossoy et al., 1999). The main function of EBP is to convert 8,9 choletenol into lathosterol, participating in the final steps of post-squalene cholesterol biosynthetic pathway (Silve et al., 1996; Derry et al., 1999; Soo-Han et al., 2001; Guggenberger et al., 2007; Ačimovič and Rozman, 2013). It has been proved that mutation of the EBP can impair cholesterol biosynthesis and cause X-chromosomal dominant chondrodysplasia punctate (Silve et al., 1996; Fabian et al., 2003). As an important functional factor in cholesterol synthesis, *EBP-like* is still an predicted novel gene in chickens. To the best of our knowledge, this was the first systematically study on the coding sequence, evolutionary origin, expression profile, regulatory mechanism, and biological functions of *EBP-like* in chickens.

It has been demonstrated in previous researches that the amino acid sequences of EBPs among mammals are highly similar. The amino acid sequence of EBP in human showed 78 and 73% identity to the amino acid sequence of EBPs in mouse and guinea pig, respectively (Moebius et al., 1998; Larios, 2014). In the present study, we found that chicken EBP-like protein sequence showed a positive identity of 65% to the amino acids of human EBP protein. It is known that EBP protein has emopamil binding domains, including the sterol acceptor site and the catalytic center, which have Delta8-Delta7 sterol isomerase activity, the functional domain in EBP protein is involved in the metabolism of sterol and long-chain fatty acids (Seo et al., 2001; Iwamoto, 2019). Here, the EBP functional domain was found in chicken EBP-like.

As reported previously, orthologous genes might be differentiated from a common ancestral gene (Yang et al., 2019). To further evaluate the evolutionary relationships between *EBP*/*EBP-like* in different species, here, a rooted evolutionary time tree was constructed with zebrafish EBP served as an outgroup sequence. The time-scale tree can help to estimate rates of molecular change in organisms and to interpret macroevolution patterns (Kumar and Hedges, 1998). The present results showed, the duplications of zebrafish and Chinese turtle *EBP* genes occurred at the earliest time, prior to the divergence of *EBP-like* genes in fish, chickens, and mammals. Duplications of mammalian *EBP* genes happened posterior to mammalian *EBP-like* genes with a large evolutionary distance of more than 90 million years. It is known that the ancestral bird lineage branch split from between avian and lizards ∼285 million years ago (Nam et al., 2010). In birds, *EBP-like* was absent in Japanese quail genome, and *EBP* was absent in chicken genome. Here, the chicken *EBP-like* and Japanese quail *EBP* gene originated from a common ancestor, suggesting a possibility of *EBP-like* replacing the missing *EBP* gene in chicken genome.

To further evaluate the sequence similarity between EBP/EBP-like in different species, the unrooted phylogenetic tree was built based on their amino acid sequences. The phylogenetic analysis results indicated that EBP/EBP-like from different species were classified into three main clusters and members in different clusters were likely to possess diverse functions. The lengths of the branches indicate the rates of similarities between sequences. According to the phylogenetic tree, EBP and EBP-like in fish, reptile, and mammals split into separate clusters, respectively. While in avian, instead of clustered with other EBP-like in mammals or fish, chicken EBP-like was classified into a distinct group with Japanese quail EBP, and showed a highly close sequence similarity relationship with EBP from other species.

Genomes are shuffled over the evolutionary timescale, so local gene-order conservation can reflect the functional constraints within the protein, which makes conservation of gene order across species crucial in predicting protein function (Huynen et al., 2000; Suyama and Bork, 2001). Here, we use comparative gene mapping to reveal the conservative synteny relationship between loci surrounding of the *EBP-like* gene in chickens and *EBP/EBP-like* gene in other species according to current genomic sequence databases. The results showed that the synteny relationships between *EBP-like* genes in human, mouse, rat, pig, and Chinese turtle were highly conservative. While chicken *EBP-like* showed no synteny relationship with any *EBP-like* in other species, but a certain level of synteny with *EBP* in other species. The *EBP-like* gene located at chicken chromosome 30, and genes (*SLC38A5*, *FTSJ1*, *PORCN*, *RBM3*, and *WAS*) that were highly conservative in mammalian chromosome X were absent in chickens. Meanwhile, *WDR13*, *SUV39H1*, and *TBC1D25*, which were conservative downstream genes of *EBP* in mammals, were located at upstream of *EBP-like* in chicken with an inconsistent order. It indicated that early rearrangement events happened in the region as vertebrate evolution. Gene rearrangement and loss are fair common phenomenon through the genome evolution. Rearrangements include inversions of genes, single-gene insertions and deletions (indels) (Crombach and Hogeweg, 2007). Substantial gene loss has occurred in all phylogenetic lineages. Gene loss can be the result of gene deletion or oblation, sequence divergence occurs from point mutations, or small deletions and insertions (Snel et al., 2002; Krylov et al., 2003; Mirkin et al., 2003; Babin, 2008; Albalat and Cañestro, 2016). It has been proved that human sterol isomerase is a homolog of mouse EBP (Seo et al., 2001). In our study, comparison among the mammals genomes demonstrated that *EBP* gene had preserved the genomic structure with no major inter-chromosomal rearrangements, indicating a better conservation of synteny of *EBP* genes along with the evolutionary process. Moreover, the consensus map revealed a relative conserved synteny between chicken *EBP-like* and *EBP* genes in other species with certain level of subgenome rearrangement.

Taken together the results of sequence alignment, functional domain analysis, rooted evolution tree, unrooted phylogenetic tree and the conserved synteny analysis, the data supported our hypothesis that *EBP-like* gene was performing the same role of mammalian *EBP* gene in chickens.

The expression of *EBP* was previously detected in a wide variety of tissues in guinea pig, including liver, ileum, colon, kidney, adrenal gland, testis, ovary, and uterus, the tissue distribution of EBP mRNAs was ubiquitous but specially abundant in liver (Silve et al., 1996). Research on rat found that *EBP* mRNA was only detected in liver and hepatocytes, not in connective tissue or blood vessels, and it remained the same expression pattern through lifetime (Soo-Han et al., 2001). Moreover, EBP protein was also reported expressed in a liver-specific manner in rat (Gaylor, 1981; Kang et al., 1995; Moebius et al., 1997; Cho et al., 1998; Soo-Han et al., 2001). In our study, *EBP-like* gene was found wildly expressed in lung, liver, spleen, kidney, glandular stomach, pectoral muscle, pancreas, duodenum, leg muscle and ovary of peak laying hens, but with a significant high abundance in liver tissue. In order to formulate eggs, peak-laying hens have an significantly high physiological cholesterol requirement, while almost all of the cholesterol in egg is come from liver through *de novo* synthesis (Liu et al., 2010). To better understand the expression characteristics of *EBP-like*, we further detected the expression levels of *EBP-like* in liver of chicken at different developmental stages. The results showed that, the expression level of *EBP-like* decreased from 5 to 20 weeks, followed by a significantly increase from 20 to 35 weeks. The reason that *EBP-like* expression dropped from 5 to 20 weeks is not quite clear, but the previous studies did show a low cholesterol level in pre-laying stage comparing with laying stage among different strains of hens (Neill et al., 1977; Bhatti et al., 2002). With the start of laying, a sharp raised *EBP-like* expression level in liver was observed from pre-laying stage (20 weeks old) to peak-laying hens (30 weeks old). A same rising trend of cholesterol levels was previously found in laying hens (Tian et al., 2019). The present results were consistent with previous studies and further supported our hypothesis that EBP-like playing the same role in cholesterol biosynthesis in chickens.

In avian, estrogen promotes the sexual maturity, and plays an essential role in the maintaining of reproductive activity by regulating the lipid metabolism of hens during the laying stage. The estrogen concentration is one of the most significant physiological changes during the peak laying period. Previous studies reported that, compared with pre-laying stage, serum estrogen level of peek-laying hens was significantly increased (Williams and Harvey, 1986). To further explore the possible reasons of EBP-like upregulated in peak-laying stage, we detected the effect of estrogen on the expression level of EBP-like. As a primary female sexual steroid, 17β-estradiol is an important hormone regulating reproduction, hepato-biliary secretion and other metabolic processes (Wang et al., 2009). Therefore, 17β-estradiol was used to simulate the chicken estrogen treatment model on both liver tissues and liver cells. It is well-studied that estrogen can increase the rate of lipoproteins synthesis and cause a significant rise in the plasma VLDL (very low-density lipoproteins) level and upregulate the levels of Apo-VLDLII mRNA. As a marker gene of estrogen treatment, Apo-VLDLII has a positive respond to 17β-estradiol (Hillyard et al., 1956; Chan et al., 1976; Hermann et al., 2003). Therefore, the expression level of Apo-VLDLII was measured *in vitro* and *in vivo* to evaluate the effects of estrogen treatments. The results showed that *EBP-like* expression level was significantly increased with the treatment of 17β-estradiol in chicken hepatocellular carcinoma cells and in chicken liver tissue. Accordingly, our results (at least partly) explained the increase of EBP expression at peak-laying stage.

Cholesterol homeostasis is regulated and maintained by three interrelated feedback mechanisms: regulation of LDL receptor production, regulation of HMGCR and other enzymes, and regulation of cholesterol 7α-hydroxylase in bile acid synthesis (Russell, 1992). As a cholesterol synthesis marker, HMGCR catalyzes the third step in the mevalonate pathway, which is known as the rate-limiting step in cholesterol synthesis (Thomas and Fell, 1998; Sharpe and Brown, 2013). DHCR7 catalyzes the final reaction of the cholesterol biosynthetic pathway (Jiang et al., 2010), mutation of it can cause the inborn error of cholesterol synthesis (Porter and Herman, 2011). EBP can interact with DHCR7 forms a heterooligomeric complex, acting as the regulatory and catalytic subunits of the antiestrogen binding site (AEBS) (Medina et al., 2010; Prabhu et al., 2016). Impairing DHCR7 activity can lead to the increase of 7-dehydrocholesterol (7DHC) and the decrease of cholesterol levels. SC5D catalyzes the conversion of lathosterol to 7DHC. Impaired SC5D activity can cause a similar deficiency of cholesterol (Jiang et al., 2010). It has been reported that DHCR7 mutant mouse embryos and SC5D mutant mouse embryos both showed a decreased cholesterol level (Krakowiak et al., 2003). DHCR24, identified as an FAD-dependent oxidoreductase in humans that reduces the Δ^24(25)^ bond of desmosterol to yield cholesterol (Waterham et al., 2001), expressed in all cells and tissues that synthesize cholesterol with the highest expression in liver (Zerenturk et al., 2013). DHCR24 also known as lanosterol reductase or desmosterol reductase. Lanosterol is the first sterol produced in the cholesterol synthetic pathway, and the Δ^24(25)^ reduction of desmosterol catalyzed by DHCR24 is the final reaction in the pathway (Soo-Han and Young-Ki, 1997; Zerenturk et al., 2013). In summary, HMGCR, SC5D, DHCR7, and DHCR24 are essential factors in the cholesterol synthetic pathway.

Cholesterol synthesis is catalyzed by a group of microsomal enzymes and reductase. The increase of expression level of the rate-limiting gene can affect the whole group of genes in the cholesterol biosynthetic pathway. For instance, it was previous proved that overexpression of sterol regulatory element-binding proteins (SREBPs) can efficiently regulate cholesterol synthesis by controlling all the enzyme genes in the cholesterol synthetic pathway (Sakakura et al., 2001; Horton et al., 2002b). However, cholesterol biosynthetic pathway is composed of complicated steps and could be under a negative feedback regulation as well. If an intermediate product in the rate-limiting step accumulates because of relative slow rate of the next reaction, instead of activating the reaction, the accumulated molecule could further downregulate the previous steps, causing a cascade of repression and even impairing the whole reaction (Sakakura et al., 2001).

In the current study, overexpression of *EBP-like* has no effect on the intracellular T-CHO level, while inhibition of the *EBP-like* expression could significantly decreased the intracellular T-CHO level. After checking the expression levels of key genes involved in cholesterol synthetic pathway, it was found that the expression levels of the *EBP-like* downstream genes (*SC5D*, *DHCR7*, and *DHCR24*) did not change with overexpression of *EBP-like*, but were significantly reduced with *EBP-like* inhibition. However, the expression levels of *HMGCR*, the upstream gene of *EBP-like*, showed no significant change of expression level when *EBP-like* was either overexpressed or inhibited. It is because that, *HMGCR* is an upstream gene of *EBP-like* in the cholesterol synthesis pathway, the expression level of *HMGCR* is not affected by the presence or the absence of *EBP-like*. Meanwhile, as a well-known rate-limiting enzyme in cholesterol synthesis pathway, HMGCR catalyzes the initial step in the cholesterol biosynthetic pathway, the rates of following reactions are largely determined by HMGCR, therefore, the expression levels of *SC5D*, *DHCR7* and *DHCR24* did not change when *EBP-like* was overexpressed. It is also possible that, the accumulation of *EBP-like* has activated the negative feedback regulation of the cholesterol biosynthesis in order to maintain a tight control of cholesterol homeostasis (Davis et al., 2004). Although overexpression of *EBP-like* gene does not change the expressions of key genes in cholesterol synthetic pathway or the intracellular T-CHO level, as an indispensable step of cholesterol biosynthesis, the absence of *EBP-like* can interfere the intracellular T-CHO synthesis through depressing the expression levels of downstream genes (*SC5D*, *DHCR7* and *DHCR24*) in cholesterol synthetic pathway. These findings suggest that *EBP-like*, without being a rate-limiting enzyme, plays an indispensable role in the process of chicken liver cholesterol synthesis.

## Conclusion

This study for the first time verified the *EBP-like* gene in chickens, and revealed its evolutionary relationship, expression pattern, regulatory mechanism and biological functions. The *EBP-like* gene was highly expressed in liver of laying hens, and its expression level significantly increased in peak-laying stage (30-weeks-old). The expression of *EBP-like* was upregulated by estrogen both *in vitro* and *in vivo*. The absence of *EBP-like* can significantly inhibit the synthesis of intracellular T-CHO by depressing the expressions of downstream genes in the cholesterol synthetic pathway. The present results indicate that, *EBP-like* gene, as a substitute of *EBP* gene in chickens, plays an essential role in the process of liver cholesterol synthesis, although it is not a rate-limiting enzyme.

## Data Availability Statement

The original contributions presented in the study are included in the article/Supplementary Material, further inquiries can be directed to the corresponding author/s.

## Ethics Statement

The animal study was reviewed and approved by the Institutional Animal Care and Use Committee (IACUC) of Henan Agricultural University Zhengzhou, China.

## Author Contributions

XL and ZM designed the project. YW and YT contributed to the literature search. KJ, ZM, and ZW performed the experiments. KJ wrote the original manuscript. XL, DL, and HL reviewed and edited the draft. All authors contributed to the article and approved the submitted version.

## Conflict of Interest

The authors declare that the research was conducted in the absence of any commercial or financial relationships that could be construed as a potential conflict of interest.

## Footnotes

1 https://blast.ncbi.nlm.nih.gov/Blast.cg

2 https://www.genomicus.biologie.ens.fr/genomicus-99.01/cgi-bin/search.pl

3 http://smart.embl-heidelberg.de/
