# Supplementary material for: Evolution, Expression Profile, Regulatory Mechanism, and Functional Verification of EBP-Like Gene in Cholesterol Biosynthetic Process in Chickens (Gallus Gallus)
Source: Front Genet. 2021 Jan 14;11:587546. doi: 10.3389/fgene.2020.587546 (PMC7841431; doi:10.3389/fgene.2020.587546)
Supplement: Supplementary file 1 [file Data_Sheet_1.docx]

Supplementary Material

# Supplementary Figures

**
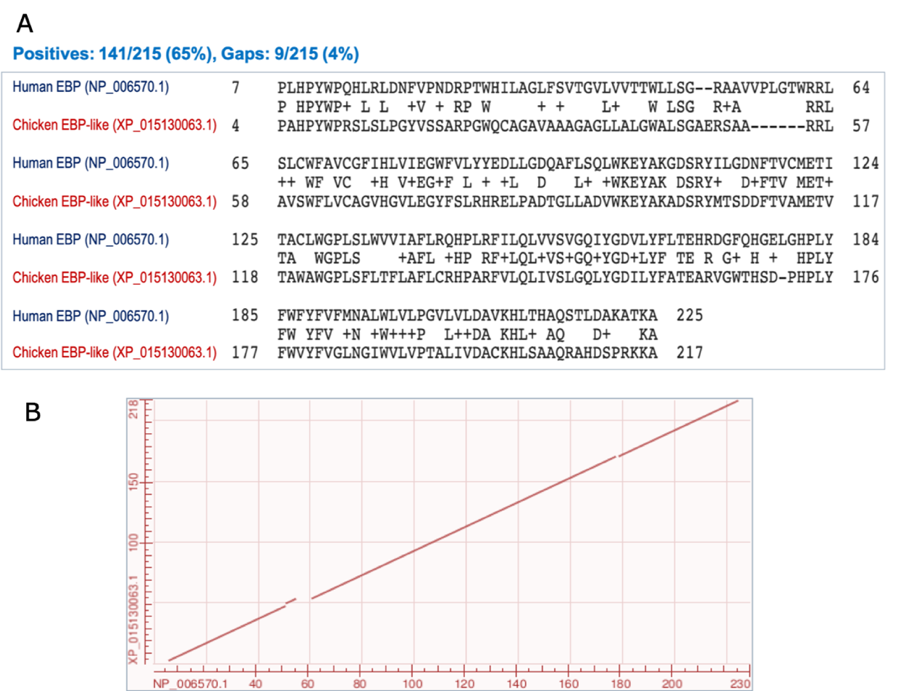
**

**Supplementary Figure 1.** Amino acid sequence alignment of human EBP protein (NP_006570.1) and chicken EBP-like protein (XP_015130063.1). (A) The alignment of proteins sequence between human EBP and chicken protein XP_015130063.1. (B) The dot matrix of amino acid sequence alignment. Note: the dot matrix shows regions of similarity based on the alignment results. The query sequence is represented on the X-axis and the subject is represented on the Y-axis. The numbers represent the residues of the subject. Alignments are shown in the plot as lines. Plus strand and protein matches are slanted from the bottom left to the upper right corner, minus strand matches are slanted from the upper left to the lower right.


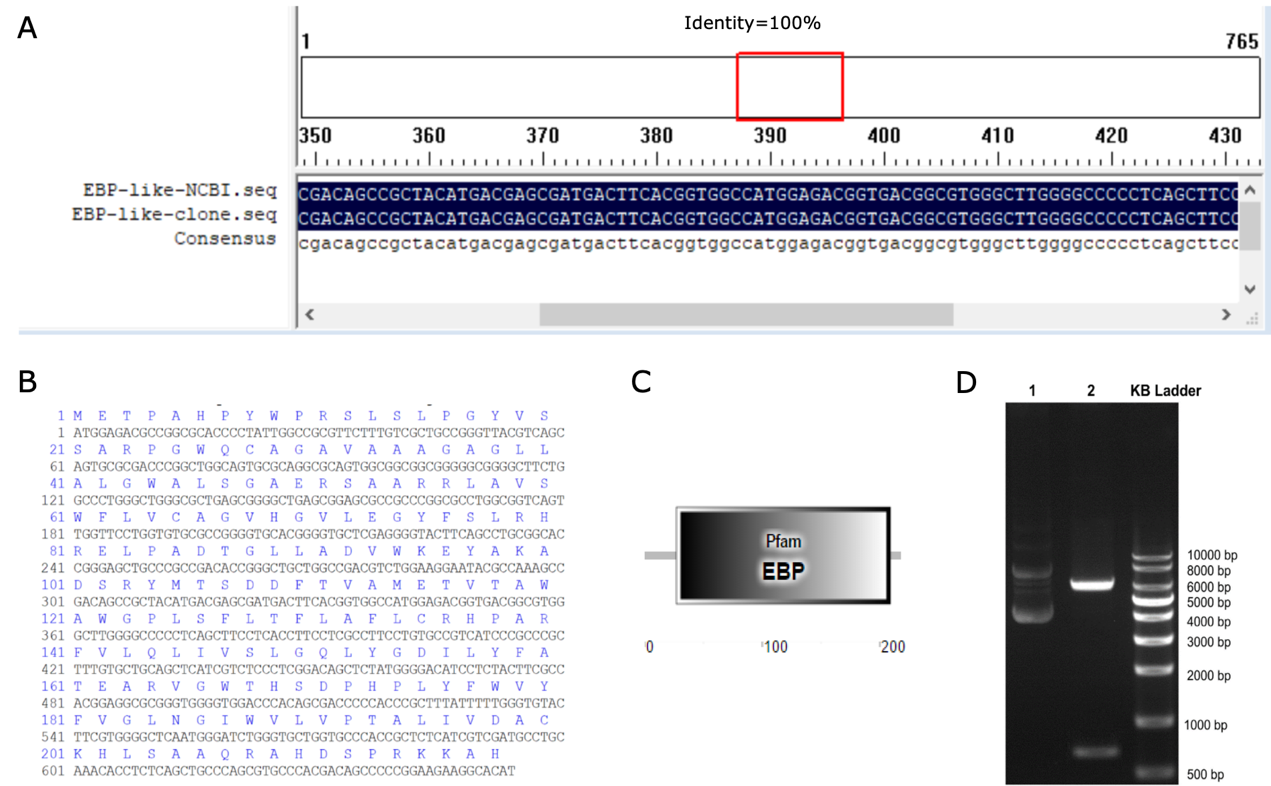


**Supplementary Figure 2.** Protein sequence analysis and chicken *EBP-like* CDs cloning. (A) Sequence alignment of cloned EBP-like CDs sequence with the *EBP-like* CDs sequence predicted on NCBI. (B) The CDs sequence and amino acid sequence of *EBP-like*. (C) Functional domain of chicken EBP-like protein. (D) The electrophoresis map after double digestion. Lane 1: pcDNA3.1-EGFP plasmid. Lane 2: pcDNA3.1-EBP-like plasmid digested by Hind III and Bam HI.

# Supplementary Table

**Supplementary table 1.** EBP/EBP-like protein sequence information in different species for phylogenetic analysis

| **Protein Name** | **NCBI Protein Sequence ID** |
| --- | --- |
| Human EBP | NP_006570.1 |
| Human EBP-like | NP_001265565.1 |
| Pig EBP | NP_001161118.1 |
| Pig EBP-like | XP_020920971.1 |
| Mouse EBP | NP_031924.1 |
| Mouse EBP-like | NP_080874.2 |
| Rat EBP | XP_010604685.1 |
| Rat EBP-like | NP_001101851.1 |
| Chicken EBP-like | XP_015130063.1 |
| Japanese quail EBP | XP_015706162.1 |
| Chinese turtle EBP | XP_006130896.2 |
| Chinese turtle EBP-like | XP_006121424.1 |
| Zebrafish EBP | NP_001002328.1 |
